# Supplementary material for: Subunit gating resulting from individual protonation events in Kir2 channels
Source: Nat Commun. 2023 Jul 28;14:4538. doi: 10.1038/s41467-023-40058-7 (PMC10382558; doi:10.1038/s41467-023-40058-7)
Supplement: Supplementary file 1 — Supplementary Information [file 41467_2023_40058_MOESM1_ESM.pdf]

Subunit gating resulting from individual protonation events in Kir2 channels

Grigory Maksaev[1,*], Michael Bründl-Jirout[2,*], Anna Stary-Weinzinger[2], Eva-Maria Zangerl-Plessl[2], Sun-Joo Lee[1], and Colin G. Nichols[1,#]

SUPPLEMENTARY DATA

Level	P_{open}	Transitions sum	Transitions fraction
4	0.59	283	0.48
3	0.26	190	0.32
2	0.07	61	0.10
1	0.02	51	0.09
0	0.06		
Total	1.00	585	1.00

Table S1. Statistical analysis of brief complete closures of cKir2.2-G178D channel at pH 7.4. Direct transitions from the closed state (Level 0) to any sub-conductive state (Levels 1-4) and direct transitions from sub-conducting states to the closed states are summarized.

Level	pH 6.5	pH 6.8	pH 7.1	pH 7.4	pH 7.7
4	7.52	7.10	7.42	7.22	7.30
3	6.56	6.53	6.68	6.79	6.88
2	5.20	5.31	5.60	5.35	5.48
1	3.79	3.72	3.91	3.30	3.81
0	0	0	0	0	0

Table S2. Amplitudes (pA) of cKir2.2-G178D mutant conducting levels at different pH. Each value is based on analysis of at least 1 min long trace.

	chain A	chain B	chain C	chain D
D173	6.14	6.76	5.73	6.84
G178D	6.34	5.89	6.35	5.23

Table S3. pKa prediction of ionizable residues in the central cavity.

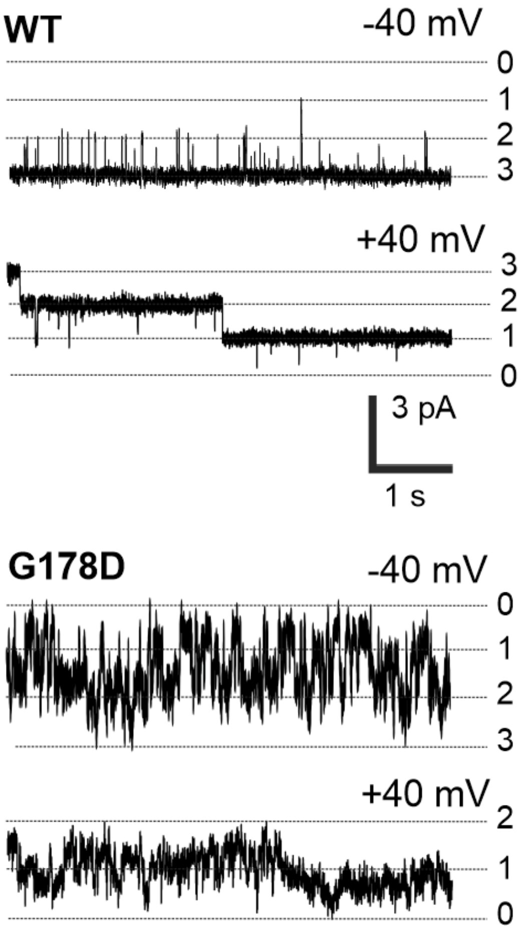

Fig. S1. Channel behavior at positive voltages Control Kir2.2 channel patch containing 3 active channels shows full conductance events only at both -40mV and at +40mV, prior to rundown. In contrast, Kir2.2[G178D] mutant channels show evidence of similar sub-conductance behavior at both -40mV and +40 mV.

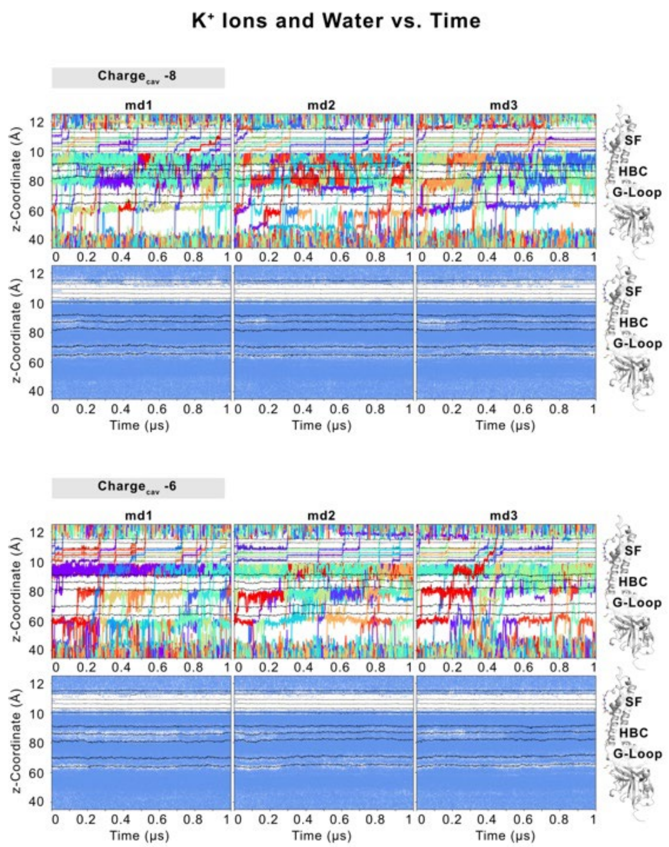
K⁺ Ions and Water vs. Time
Charge_cav -8
Charge_cav -6
md1
md2
md3
z-Coordinate (Å)
Time (μs)
SF
HBC
G-Loop

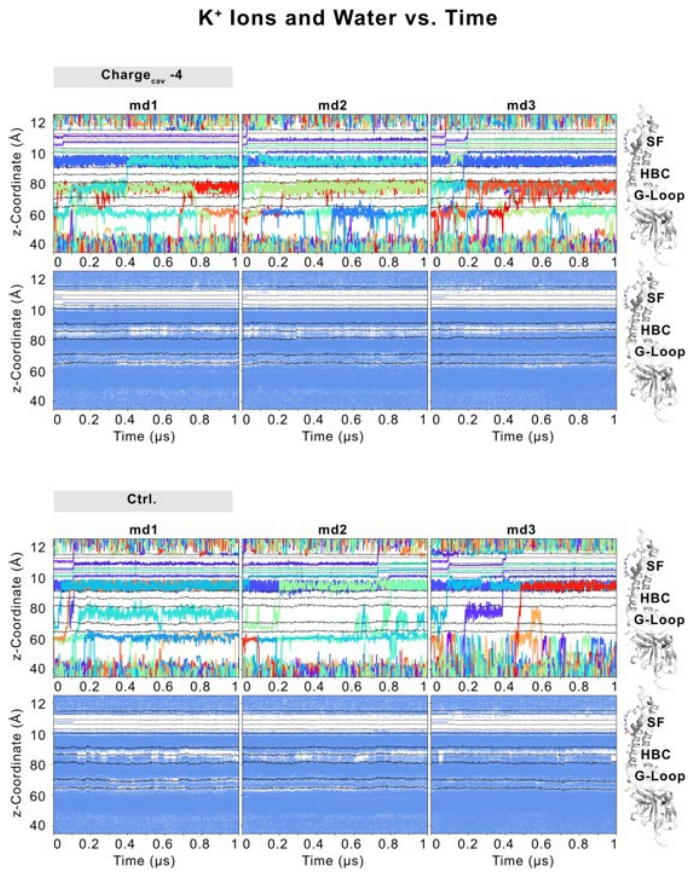

Figure S2. The flux of individual K^+ ions through the channel pore (z-coordinate of the simulation box) for repeated simulations of different constructs.

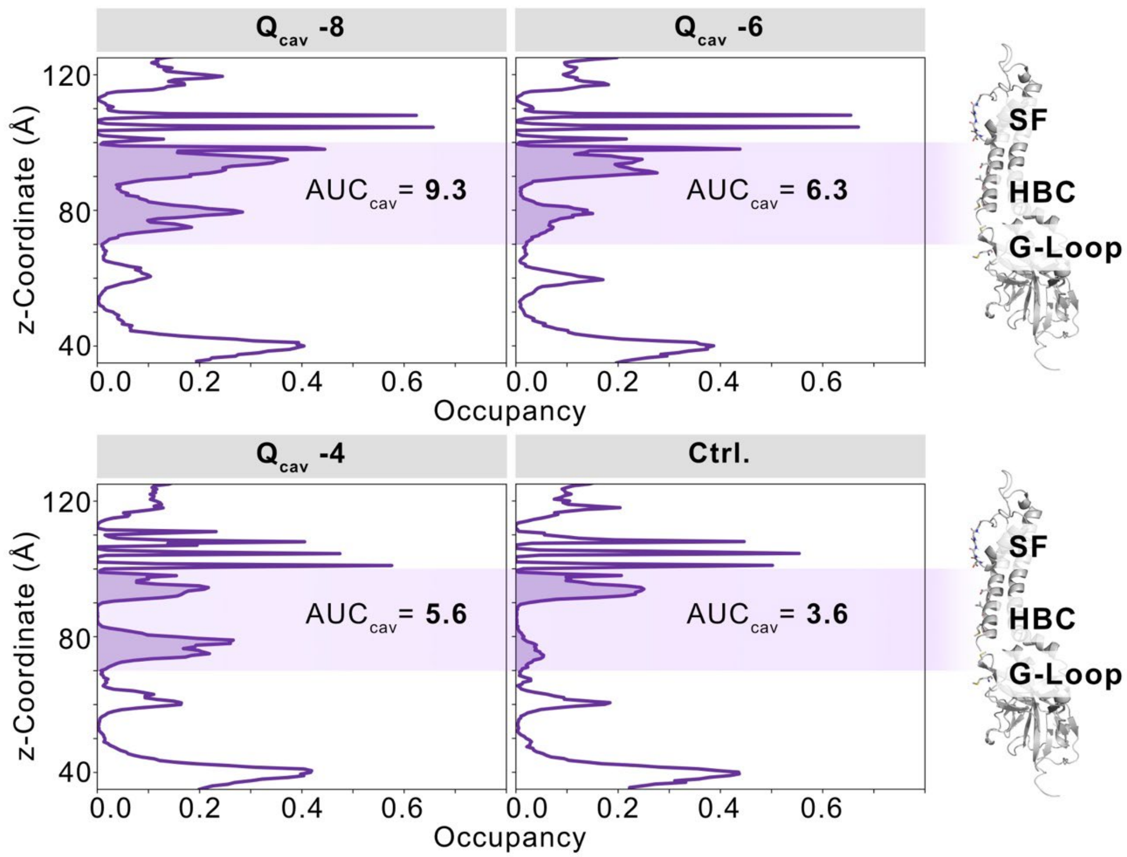

Figure S3. K^+ occupancy increases with number of deprotonated residues in the pore. AUC_{cav} shows the number of K^+ ions in the pore between the lower SF and M308 of the G-loop gate, each averaged over three replicas per system.

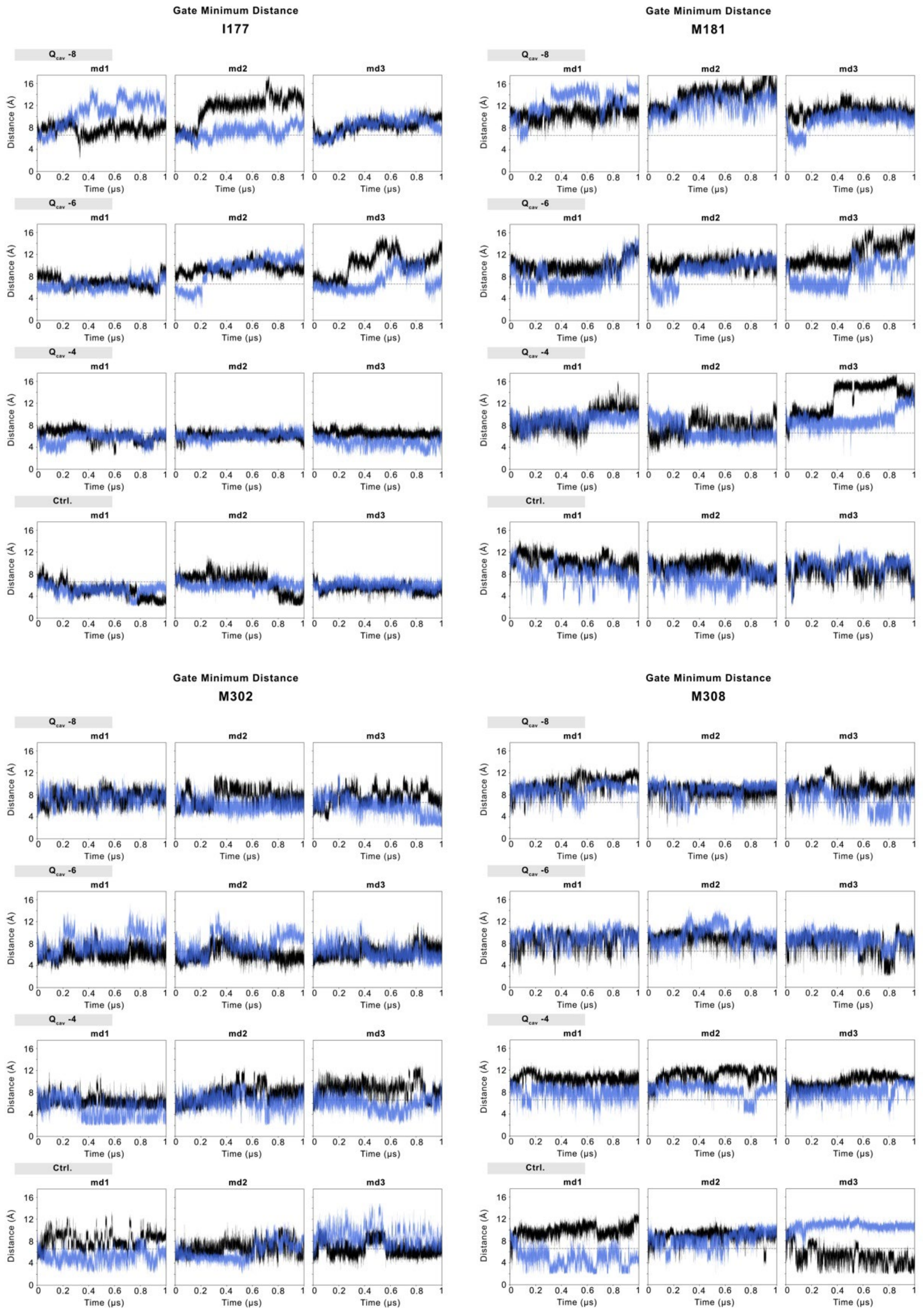

Figure S4. Minimum distances of gating residues over time between two opposing subunits. The corresponding histograms can be found in Fig. 7.
