# Supplementary material for: Subunit gating resulting from individual protonation events in Kir2 channels
Source: Nat Commun. 2023 Jul 28;14:4538. doi: 10.1038/s41467-023-40058-7 (PMC10382558; doi:10.1038/s41467-023-40058-7)
Supplement: Supplementary file 3 — Description of Additional Supplementary Files [file 41467_2023_40058_MOESM3_ESM.pdf]

Description of Additional Supplementary Files

File Name: Supplementary Movie 1
Description: **md1 of system Cav_{charge} -8.** Ion Flux and Pore Solvation. Two opposing subunits of the protein are shown, while relevant residues are visualized in stick representation. To track ion flux, K^+ ions are represented as spheres and colored uniquely (the colors for K^+ ions do not correspond with those in Figure 1). A transparent surface around water molecules in the pore illustrates pore solvation.

File Name: Supplementary Movie 2
Description: **md1 of system Cav_{charge} -4.** Ion Flux and Pore Solvation. Two opposing subunits of the protein are shown, while relevant residues are visualized in stick representation. To track ion flux, K^+ ions are represented as spheres and colored uniquely (the colors for K^+ ions do not correspond with those in Figure 1). A transparent surface around water molecules in the pore illustrates pore solvation.
